# The Distribution and Survival Association of Genetic Polymorphisms in Thai Patients with Hepatocellular Carcinoma According to Underlying Liver Disease

**DOI:** 10.3390/genes16070808

**Published:** 2025-07-09

**Authors:** Theint Cho Zin Aung, Bootsakorn Boonkaew, Maneerat Chayanupatkul, Kittiyod Poovorawan, Natthaya Chuaypen, Pisit Tangkijvanich

**Affiliations:** 1 Division of Academic Affairs, Faculty of Medicine, Chulalongkorn University, Bangkok 10330, Thailand; 6678004630@student.chula.ac.th; 2 Center of Excellence in Hepatitis and Liver Cancer, Department of Biochemistry, Chulalongkorn University, Bangkok 10330, Thailand; bootsakorn.b@gmail.com; 3 Division of Gastroenterology, Department of Medicine, Faculty of Medicine, Chulalongkorn University, Bangkok 10330, Thailand; maneeratc@gmail.com; 4 Department of Clinical Tropical Medicine, Faculty of Tropical Medicine, Mahidol University, Bangkok 14000, Thailand; kittiyod.poo@mahidol.ac.th; 5 Metabolic Disease in Gastrointestinal and Urinary System Research Unit, Department of Biochemistry, Faculty of Medicine, Chulalongkorn University, Bangkok 10330, Thailand; natthaya.c@chula.ac.th

**Keywords:** hepatocellular carcinoma, steatotic liver disease, polymorphisms, *PNPLA3*, *TM6SF2*, *HSD17B13*

## Abstract

Background/Objectives: The influence of single-nucleotide polymorphisms (SNPs) on hepatocellular carcinoma (HCC) in terms of etiological factors remains to be explored. This study evaluated the distribution of *PNPLA3* rs738409, *TM6SF2* rs58542926, and *HSD17B13* rs6834314 and overall survival of HCC patients with metabolic dysfunction-associated steatotic liver disease (MASLD-HCC) and viral-related HCC (VIRAL-HCC). Methods: This study included 564 patients with HCC: 254 with MASLD-HCC and 310 with VIRAL-HCC. The SNPs were determined by real-time PCR using TaqMan assays. Results: The mean ages of patients with MASLD-HCC and VIRAL-HCC were 68.4 vs. 60.9 years (*p* < 0.001), with a significant difference between groups. The prevalence of *PNPLA3* GG genotype in MASLD-HCC was significantly higher in MASLD-HCC than in VIRAL-HCC (24.0% vs. 15.5%, OR = 1.86, 95% CI = 1.14–3.05, *p* = 0.009). Similarly, the prevalence of *TM6SF2* TT genotype in MASLD-HCC and VIRAL-HCC was 7.1% vs. 2.6% (OR = 3.39, 95% CI = 1.36–9.21, *p* = 0.003), while *HSD17B13* GG genotype in the corresponding groups was 7.1% vs. 12.6% (OR = 0.53, 95% CI = 0.27–1.01, *p* = 0.039). The overall median survival of MASLD-HCC was significantly shorter than that of the VIRAL-HCC group (42 vs. 66 months, *p* = 0.035). In Cox regression hazard analysis, *HSD17B13* GG genotype was significantly associated with a lower mortality rate in MASLD-HCC (HR = 0.38, 95% CI = 0.18–0.81, *p* = 0.011). In contrast, *PNPLA3* and *TM6SF2* were not associated with overall survival in patients with MASLD-HCC or VIRAL-HCC. Conclusions: Our data demonstrated that the prevalence of the SNPs significantly differed between MASLD-HCC and VIRAL-HCC. The *HSD176B13* GG genotype was also associated with a survival benefit in Thai patients with MASLD-HCC. Thus, assessing the *HSD176B13* genotype might be beneficial in risk stratification and potential therapeutic inhibition of *HSD17B13* among patients with MASLD-HCC.

## 1. Introduction

Hepatocellular carcinoma (HCC) represents the most aggressive and common liver cancer, representing approximately 90% of all cases on a global scale [1]. Notably, the HCC incidence ratio of men to women is approximately 3:1, with a 5-year survival rate of 20%, as patients have been diagnosed at advanced stages when curative treatment is not possible [2]. HCC commonly develops under the backdrop of chronic liver disease, cirrhosis with diverse etiologies: Hepatitis B Virus (HBV), Hepatitis C Virus (HCV), excessive alcohol consumption, genetic risks, metabolic dysfunction-associated steatotic liver disease (MASLD) [3]. Accordingly, the distribution of underlying etiological factors alters depending on the epidemiological patterns and underlying cirrhosis [2]. Although chronic HBV infection is the key risk factor, accounting for over 50% of HCC cases [2], HBV vaccination has remarkably reduced the prevalence of infection and the incidence of HCC globally [4]. Additionally, the accessibility of direct-acting antiviral agents (DAAs) decreases the worldwide burden of HCC-related incidence and mortality [5]. Apart from the viral infection, non-infectious risk factors leading to HCC occurrence have alarmingly increased, characterized by abnormal metabolic functions [6].

MASLD, previously known as non-alcoholic fatty liver disease (NAFLD), is diagnosed by the existence of hepatic steatosis (≥5%), complemented by at least one cardiometabolic disorder [7]. MASLD is a multi-system disorder connected to metabolic syndrome, including type 2 diabetes mellitus, insulin resistance, obesity, dyslipidemia, and hypertension [7]. The progression of HCC in MASLD is complicated, originating from fat deposition in hepatocytes and associated lipotoxicity, fibrogenesis, hepatocyte regeneration, and genetic mutations, which lead to an increased tendency of HCC development [7]. The overall global prevalence of MASLD-HCC is 30% [8], with a regional rating of 33% in southeast-Asian Countries, including Thailand [9]. Interestingly, the corresponding rates for MASLD-HCC in females become almost parallel to the male approximations [10]. Even though cirrhosis leads to a 10-fold increase in HCC development, it is worth noting that approximately 20% of MASLD-HCC cases arise at an early stage of HCC, regardless of cirrhosis [11].

Several recent studies have indicated the role of single-nucleotide polymorphisms (SNPs) in the development and progression of MASLD [12,13]. Among them, the *patatin-like phospholipase domain-containing protein 3 (PNPLA3)* gene displays an essential role in lipid metabolism by regulating the rate-limiting step in triglyceride hydrolysis [14]. Indeed, the *PNPLA3* rs738409 polymorphism represents one of the strongest and most consistent SNPs related to increased risk of MASLD progression, which potentially could lead to HCC development [15]. The *transmembrane 6 superfamily member 2 (TM6SF2)* gene, functioning in lipid metabolism, is also related to an increased risk of MASLD progression [16]. The minor allele of the *TM6SF2* rs58542926 variant could lead to severe hepatic steatosis progression in terms of impaired lipid metabolism, and its association is independent of *PNPLA3* rs738409’s effect [17]. An additional SNP associated with MASLD is the variant in the *Hydroxysteroid 17-beta dehydrogenase 13 (HSD17B13)* gene, which plays a key role in retinoid metabolism through its enzymatic activity and catalyzing retinol oxidation to retinaldehyde [18]. Interestingly, the loss-of-function *HSD17B13* variant has been recognized as a hepatoprotective factor of MASLD, and this *HSD17B13* rs6834314 G allele was independently correlated with decreasing incidence of liver-related consequences [19]. Although these SNPs are involved in the pathogenesis of MASLD, it remains uncertain whether they are related to the development and prognosis of HCC, considering different underlying liver diseases. Therefore, it would be necessary to explore the impact of the SNPs on cancer development in patients with related HCC (MASLD-HCC) and virus-related HCC (VIRAL-HCC).

In this study, we aimed to investigate the potential impact of these genetic variants on cancer development among patients, specifically concerning the etiological factors of HCC. In addition, we determined whether these SNPs influenced the overall survival of individuals with MASLD-HCC compared to those with VIRAL-HCC.

## 2. Materials and Methods

### 2.1. Study Population

This study comprised 564 HCC participants, recruited after a radiologic- and/or pathologic-confirmed diagnosis of HCC from King Chulalongkorn Memorial Hospital (Bangkok, Thailand) between 2014 and 2024. Among them, 254 participants were MASLD-HCC, and 310 patients were VIRAL-HCC [228 with chronic HBV infection (HBV-HCC), and 82 with chronic HCV infection (HCC-HCV)]. Regarding the MASLD-HCC group, the diagnosis was based on evidence of the presence of liver steatosis alongside a clinical phenotype of metabolic dysfunction without seropositivity for HBsAg and anti-HCV. Additionally, 180 healthy individuals without any liver disease were included as the control group (“Healthy controls”). Blood samples that had been collected were separated, and then DNA was extracted and stored at −80 °C. This research had received the approval of the Institutional Review Board (IRB No. 0585-67) and was conducted following the Declaration of Helsinki. All the participants had provided written informed consent, and all the methods were carried out according to the standard guidelines.

### 2.2. Diagnosis and Follow-Up of HCC

According to the recommendation of the American Association for the Study of Liver Disease (AASLD), the diagnosis of HCC was conducted using standard imaging investigations and/or histology. Diagnosing criteria of HCC were based on specific lesions with hyperattenuation at the arterial phase and hypoattenuation at the portal phase in dynamic Computed Tomography (CT) scans or Magnetic Resonance Imaging (MRI) [20]. If typical imaging features were inconclusive, liver biopsy or fine needle aspiration was further investigated. Patients’ demographic data, such as age, gender, BMI, and clinical information, including tumor size, extrahepatic metastasis, ascites, and laboratory information, were recorded. Cirrhosis was defined using the Child–Pugh score, while HCC staging was categorized based on the Barcelona Clinic Liver Cancer staging system (BCLC) [21]. After being diagnosed with HCC, the patients were treated with appropriate modalities, including surgical resection, liver transplantation, local ablative treatment, systemic therapies, and supportive care. These treatments depended on various factors, including HCC stages and the patient’s overall health. The follow-up period was performed at the diagnostic date of HCC, until either the date of death or the complete registration date, between September 2014 and January 2025.

### 2.3. DNA Preparation and SNP Genotyping

Genomic DNA was isolated from 100 μL of peripheral blood mononuclear cells (PBMCs) utilizing the phenol-chloroform-isoamyl alcohol technique. DNA quality was assessed by a spectrophotometer (NanoDrop 2000c, Thermo Scientific, Wilmington, DE, USA). The three polymorphisms, PNPLA3, TM6SF2, and HSD17B13, were analyzed using TaqMan real-time PCR protocols. The reaction mixture contained 4 µL of 2.5× master mix (5 PRIME, QIAGEN Beverly Inc., Beverly, MA, USA), 0.25 µL of 40× primer and probe mixture from the TaqMan SNP Genotyping Assay (assay ID: C_7241_10, Applied Biosystems, Waltham, MA, USA), 50–100 ng of genomic DNA, and nuclease-free water, reaching a total volume of 10 µL. Following this, real-time PCR was carried out on a QuantStudio3 Real-time PCR system (Applied Biosystems, USA). The process involved a denaturation step at 95 °C for 10 min, followed by 50 amplification cycles comprising denaturation at 92 °C for 10 s and annealing/extension at 60 °C for 1 min. Fluorescent signals (FAM and VIC) were recorded at each cycle’s end. Each experiment included positive and negative controls to interpret data accuracy. The allelic discrimination plot was evaluated using the QuantStudio^TM^ 3 Real-Time PCR System (Thermo Fisher Scientific, USA).

### 2.4. Statistical Analysis

Statistical analysis was performed using the STATA software (version 18.0) for Mac (College Station, TX, USA, Stata Corp. 2023). A comparison between groups was analyzed using the Chi-square or Fisher’s exact test for the categorical variables, which were presented as frequencies and percentages. In addition, the Student’s *t*-test or Mann–Whitney U test was applied to analyze continuous variables, with mean ± standard deviation (SD). The Hardy–Weinberg equilibrium (HWE) was determined using Pearson’s Chi-Square to evaluate the distribution of allele and genotype frequencies. The odds ratio (OR) with a 95% confidence interval (CI) between the two study groups was documented. A logistic regression analysis was conducted to assess factors associated with MASLD-HCC. Moreover, the overall survival of patients with HCC was assessed using the Kaplan–Meier and the log-rank test. Subsequently, Cox Regression was applied to evaluate independent associations between genetic variables, clinical risk factors, and mortality following the HCC diagnosis. The hazard ratio was presented with a forest plot using GraphPad Prism (GraphPad Software, Version 9.3.1, Boston, MA, USA). The *p*-value < 0.05 was regarded as statistically significant.

## 3. Results

### 3.1. Patient Characteristics

Baseline characteristics, including the study population’s demographic, clinical, and laboratory features, categorized into healthy controls, the MASLD-HCC, and VIRAL-HCC groups, are summarized in Table 1. Patients with MASLD-HCC were significantly older and had higher body mass index (BMI) than the VIRAL-HCC group. Additionally, the prevalence of diabetes mellitus and hypertension was significantly higher in the MASLD-HCC group than in the VIRAL-HCC group. Compared to the VIRAL-HCC group, MASLD-HCC patients exhibited lower mean hemoglobin, aspartate aminotransferase (AST) and alanine aminotransferase (ALT) levels, but higher platelet counts. Regarding tumor characteristics, patients with MASLD-HCC had more advanced HCC at initial presentation than the other group, in terms of tumor size, BCLC stages, and extrahepatic metastasis. Nevertheless, the proportion of cirrhosis was higher in the VIRAL-HCC group compared to the MASLD-HCC group. Of note, alpha-fetoprotein (AFP) levels were higher in the VIRAL-HCC group than in the MASLD-HCC group.

### 3.2. Distribution of SNPs in Each Studied Group

The genotyping assays for the three SNPs—PNPLA3 rs738409, TM6SF2 rs58542926, and HSD17B13 rs6834314—were conducted on samples from healthy controls and all HCC patients. The genotype frequencies of each SNP across the entire cohort showed no deviations from the Hardy–Weinberg equilibrium (*p* > 0.05), as described in Table S1. The genotype and allele frequencies of SNPs in healthy controls and the two study groups are demonstrated in Table 2. The frequencies of the PNPLA3 GG and CG + GG genotypes were significantly lower in healthy controls compared to the MASLD-HCC group (OR 0.36; 95% CI 0.19–0.66; *p* < 0.001, and OR 0.59; 95% CI 0.39–0.91; *p* = 0.009, respectively), but their frequencies did not significantly differ from the VIRAL-HCC group. Similarly, the frequencies of TM6SF2 CT, TT and CT + TT genotypes were lower in healthy controls vs. the MASLD-HCC (OR 0.49; 95% CI 0.30–0.80; *p* = 0.003, OR 0.06; 95% CI 0.001–0.4; *p* < 0.001, and OR 0.41; 95% CI 0.25–0.66; *p* < 0.001, respectively). Additionally, the HSD17B13 AG, GG, and AG + GG genotypes were higher in healthy controls vs. the MASLD-HCC group (OR 1.70; 95% CI 1.11–2.62; *p* = 0.01, OR 2.52; 95% CI 1.20–5.31; *p* = 0.006, and OR 1.82; 95% CI 1.21–2.75; *p* = 0.002, respectively). The frequencies of HSD17B13 AG and AG+GG genotypes were also higher in healthy controls vs. the VIRAL-HCC group (OR 1.71; 95% CI 1.13–2.60; *p* = 0.008, and OR 1.62; 95% CI 1.09–2.41; *p* = 0.01, respectively).

The frequencies of the PNPLA3 GG genotype were significantly higher in the MASLD-HCC group, compared to VIRAL-HCC (OR 1.86; 95% CI 1.14–3.05; *p* = 0.009). Correspondingly, the frequencies of TM6SF2 CT, TT and CT+ TT genotypes were higher in the MASLD-HCC, compared with the VIRAL-HCC group (OR 1.88; 95% CI 1.24–2.83; *p* = 0.002, OR 3.39; 95% CI 1.36–9.21; *p* = 0.003, and OR 2.05; 95% CI 1.39–3.02; *p* <0.001 respectively). Furthermore, the minor allele frequency of the TM6SF2 T allele in the MASLD-HCC group was significantly higher than in the VIRAL-HCC group (OR 2.32; 95% CI 1.03–5.42; *p* = 0.027)**.** In contrast, the HSD17B13 GG genotype among MASLD-HCC patients was significantly lower than that of the VIRAL-HCC group (OR 0.53; 95% CI 0.27–0.97; *p* = 0.039), while the minor allele frequency of the HSD17B13 G allele was comparable between the two study groups.

### 3.3. Association of Factors with the Overall Survival of Patients with HCC

The mean and median follow-up times per participant were 1.8 and 1 year, respectively. The overall median survival time (MST) of HCC patients was 51 months, with MASLD-HCC significantly shorter than the VIRAL-HCC group (42 vs. 66 months, *p* = 0.035), as shown in Table 3. The overall survival of patients with HCC was significantly associated with HSD17B13 rs6834314, underlying etiologies, tumor size, and the presence of extrahepatic metastasis. However, there was no association with PNPLA3 rs738409, TM6SF2 rs58542926, age, sex, diabetes, hypertension, cirrhosis, Child–Pugh score, or BCLC stage.

**Figure 1:**
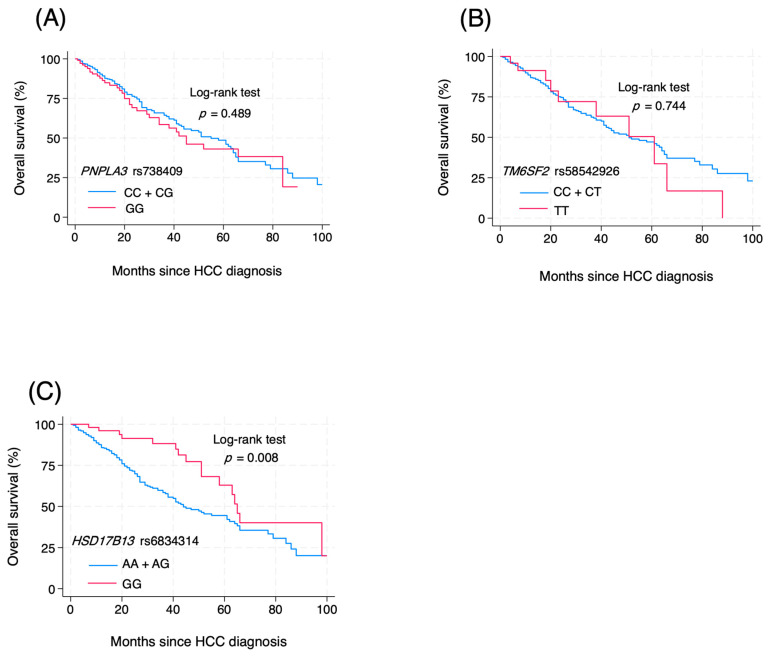
Effect of genetic polymorphisms on the prognosis of HCC (**A**) *PNPLA3* rs738409 CC + CG vs. GG; (**B**) *TM6SF2* rs58542926 CC+CT vs. TT; (**C**) *HSD17B13* rs6834314 AA + AG vs. GG.

**Figure 2:**
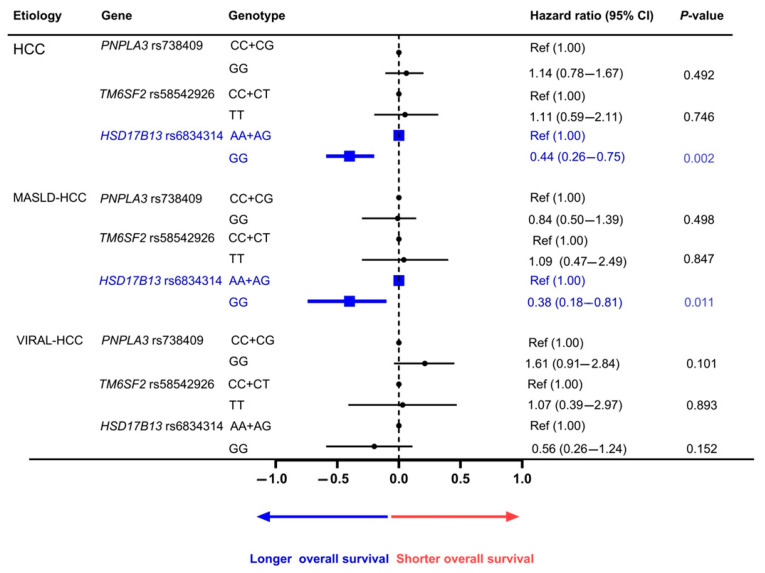
Adjusted correlation between genetic polymorphisms and overall survival of HCC.

The prognostic association of the studied SNPs was also investigated using the Kaplan–Meier method (Figure 1). For PNPLA3 rs738409, the overall survival of HCC patients carrying the CC + CG and GG genotypes was not significantly different (*p* = 0.489 by log-rank test) (Figure 1A). Similarly, for the prognostic role of TM6SF2 rs58542926, the overall survival of patients carrying the CC + CT and TT genotypes was similar in all HCC cases (*p* = 0.744) (Figure 1B). In contrast, the overall survival of patients with HCC concerning HSD17B13 rs6834314 differed significantly between patients carrying the AA + AG and GG genotypes (*p* = 0.008) (Figure 1C). Among the MASLD-HCC group, the HSD17B13 GG genotype was associated with the overall survival, while the PNPLA3 rs738409 and TM6SF2 rs58542926 did not show any association. Regarding the VIRAL-HCC group, none of the studied SNPs showed a significant correlation with overall survival (Figure 2, Figures S1 and S2).

## 4. Discussion

HCC represents the most prevalent form of primary liver cancer, comprising around 90%. The development of HCC is associated with various risk factors, which include chronic viral infections, environmental influences, genetic predispositions, and metabolic syndrome. Recent studies have indicated that MASLD is becoming increasingly common and is a growing concern. MASLD is a multi-system condition associated with several risk factors, including the influence of genetic variations that play a crucial role in the progression of advanced disease. This study aimed to evaluate the impact of the genetic polymorphisms *PNPLA3* rs738409, *TM6SF2* rs58542926, and *HSD7B13* rs6834314 on susceptibility to hepatocellular carcinoma (HCC) in a cohort of HCC patients with two different etiologies: MASLD-HCC and VIRAL-HCC, as well as to determine whether these genetic variations correlated with the prognosis of HCC. We selected these polymorphisms for analysis in this study due to their strong association with the development and progression of MASLD in Asian populations [13].

Our data demonstrated that the prevalence of the SNPs differed between MASLD-HCC and VIRAL-HCC. Specifically, *PNPLA3* GG and *TM6SF2* TT genotypes had greater distribution in patients with MASLD-HCC compared to the VIRAL-HCC group. In contrast, the *HSD17B13* GG genotype was less prevalent in the MASLD-HCC than the VIRAL-HCC group. Our results also indicated that overall survival following the diagnosis of HCC was associated with *HSD17B13* rs6834314 genotype, particularly among patients with MASLD-HCC, as individuals with the *HSD7B13* GG genotype seemed to exhibit a survival advantage compared to those with the other genotypes. Conversely, no significant evidence linked the genetic susceptibility variants in *PNPLA3* and *TM6SF2* to the overall survival of patients with MASLD-HCC and VIRAL-HCC.

Advancements in genomic research have revolutionized the approaches for identifying the genetic factors related to MASLD development. Recent studies have recognized that genome-wide and exome-wide association studies have identified associations between specific genetic variants, particularly SNPs, and the multi-factorial process of liver carcinogenesis [22]. *HSD17B13,* a member of the 17-beta hydroxysteroid dehydrogenase family, is highly expressed in the lipid droplets of hepatocytes and plays a key role in retinoid metabolism, converting retinol to retinaldehyde through its retinol dehydrogenase activity [23]. An intergenic variant, rs6834314 *HSD17B13*, triggers the substitution of adenine (A) with guanine (G), resulting in both major and minor alleles, and leading to a loss of function. Although the pathogenic role of *HSD17B13* genetic variants in MASLD has not yet been completely understood, several reports have demonstrated their relation to the clinical spectrum of MASLD.

For instance, a recent Japanese study revealed that the *HSD17B13* rs6834314 AG + GG genotypes were related to enhanced steatosis but displayed a reduced effect of *PNPLA3* on advanced fibrosis [24]. A study of the Caucasian population with biopsy-proven MASLD indicated that the G minor allele of *HSD17B13* rs6834314 was potentially related to steatosis development, and it might decrease liver inflammation and ballooning [25]. Additionally, a long-term follow-up of multi-ethnic Asian people with MASLD demonstrated that the *HSD17B13* rs6834314 SNP was linked to decreased steatotic liver disease and lower hepatic complications [19]. A recent report demonstrated that HCC patients harboring the *HSD17B13* rs72613567 TA variant displayed a survival benefit after diagnosing HCC [26], which aligned well with our report. Together, these data indicate that *HSD17B13* polymorphism could be a protective factor for MASLD progression by mitigating liver-related complications. The molecular mechanism by which *HSD17B13* variants are responsible for the hepatoprotective effects of HCC remains to be elucidated. Previous data, including histological evidence, have reported that loss-of-function variants may reduce retinol dehydrogenase activity, leading to a decrease in inflammatory and fibrogenic processes [23,25]. In this context, the potential application of inhibiting *HSD17B13* as a therapeutic approach for HCC is currently being investigated, which might be valuable in treating this aggressive cancer [27].

Notably, we did not demonstrate a correlation between the *PNPLA3* rs738409 and *TM6SF2* rs58542926 polymorphisms and overall survival in our HCC cohort. *PNPLA3* is a transmembrane protein expressed principally in the liver and displays hydrolase activity against triglycerides [28]. Thus, the loss-of-function *PNPLA3* rs738409 variant is associated with increased intrahepatic triglyceride deposition, leading to an inflammatory process, progressive liver disease, and the development of HCC [29]. A meta-analysis of Western populations has shown that this polymorphism is linked to an augmented risk of HCC in patients with MASLD and ALD [30,31]. Additionally, data in Asian patients have indicated that this variant is associated with advanced fibrosis and HCC in patients with NAFLD [32,33]. In a recent meta-analysis, a global pattern of *PNPLA3 GG* has been revealed, indicating its association with an increased risk of liver-related complications and worse clinical outcomes among patients with MASLD [34]. For instance, some studies demonstrated that the *PNPLA3* rs738409 variant might determine susceptibility to HCC development and poor prognosis after diagnosis [35,36]. In contrast, our study did not support these findings, as the SNP exhibited only the susceptibility of MASLD-HCC occurrence but had a neutral effect on overall survival. The explanation for this discrepancy was unclear, but it might suggest that the differences in ethnicities or clinical characteristics in each cohort could impact the diverse associations among reports.

*TM6SF2*, predominantly expressed in the liver and kidney, has been shown to play a key role in modulating lipid metabolism, particularly in the liver. *TM6SF2* is mainly involved in the secretion of intrahepatic triglycerides, thereby modulating the intracellular lipid content [37]. The genetic variant of *TM6SF2* rs58542926, which leads to reduced protein expression, is linked to higher intrahepatic triglyceride accumulation [38]. Of note, persons carrying the variant have exhibited an increased risk for MASLD development but a decreasing cardiovascular disease risk [39]. Like the *PNPLA3* variant, *TM6SF2* rs58542926 contributes to the progressive liver disease in patients with MASLD, initiating from simple steatosis to progressive fibrosis and cirrhosis [40]. According to a meta-analysis, the minor allele T of *TM6SF2* rs58542926 is associated with a higher risk of severe steatosis and fibrosis in children and adults [41]. Furthermore, a European cohort also indicated that the *TM6SF2* variant was significantly associated with liver-related complications in advanced liver disease [42]. The meta-analysis findings also suggest a significant association of *TM6SF2* polymorphism with HCC risk, particularly in patients with steatotic liver disease [43]. However, the available data concerning the role of this polymorphism in HCC prognosis are lacking and need further investigations.

This study had some limitations, primarily due to its case–control design, which comprised only Thai patients and may not be applicable to other ethnic populations. Moreover, this study did not analyze other genetic variants associated with MASLD, such as *MBOAT7* rs641738 and *GCKR* rs1260326. The explanations were based on a relatively low *MBOAT7* rs641738 T allele frequency in Asian people, and the role of the variant *GCKR* rs1260326 regarding MASLD development might diverge across different Asian populations [44,45]. Despite these limitations, our data suggested that the prevalence of the polymorphisms *PNPLA3* rs738409, *TM6SF2* rs58542926, and *HSD17B13* rs6834314 significantly differed between MASLD-HCC and VIRAL-HCC. Additionally, the *HSD176B13* GG genotype was associated with a survival benefit in Thai patients with MASLD-HCC after being diagnosed with the cancer. Our findings aligned with previous data indicating the consistency of the hepatoprotective effect of the *HSD17B13* polymorphism across ethnic groups. Indeed, several oligonucleotide-based agents targeting HSD17B13 are currently being developed in clinical trials, with promising outcomes [46]. Thus, genetic determination of the variant could lead to targeting HSD17B13 or modulating its activity, which may offer personalized therapeutic options for managing patients with MASLD-HCC.

## Figures and Tables

**Table 1:** Baseline characteristics of healthy controls and patients with MASLD-HCC and VIRAL-HCC.

Characteristics	Healthy Controls (*n* = 180)	MASLD-HCC (*n* = 254)	VIRAL-HCC (*n* = 310)	*p*
Age (Years)	50.0±7.6	68.1±11.7	60.9±10.5	<0.001 *
Sex (%)				<0.001 *
Male	20 (11.1)	179 (70.5)	241 (77.7)	
Female	160 (88.9)	75 (29.5)	69 (22.3179)	
Body mass index (kg/m^2^)		24.99±4.9	23.8±4.1	0.004 *
Diabetes mellitus (Yes)		98 (38.6)	36 (11.6)	<0.001 *
Hypertension (Yes)		98 (38.6)	40 (12.9)	<0.001 *
Hemoglobin (g/dL)		11.5±2.3	12.4±2.1	<0.001 *
Platelet count (10^3^/μL)		200.7±102.0	176.2±94.5	0.003 *
Total bilirubin (mg/dL)		0.9±0.6	1.0±0.9	0.041 *
AST (IU/L)		44.2±22.0	60.5±35.8	<0.001 *
ALT (IU/L)		34.2±17.8	46.9±25.9	<0.001 *
ALP (IU/L)		122.8±60.9	118.5±52.0	0.385
Albumin (mg/dL)		3.6±0.5	3.5±0.6	0.071
INR		1.1±0.1	1.2 ±0.1	<0.001 *
AFP (ng/mL)				<0.001 *
≤100		195 (76.8)	172 (55.5)	
≥100		59 (23.2)	138 (44.5)	
Tumor size (cm)		6.6 ±4.9	6.2 ±4.9	0.317
Extrahepatic metastasis (Yes)		87 (34.3)	41 (13.2)	<0.001 *
Ascites (Yes)		54 (21.3)	64 (20.6)	0.858
Cirrhosis (Yes)		182 (71.7)	260 (83.9)	<0.001 *
Child–Pugh Score				0.837
A		204 (80.3)	247 (79.7)	
B, C		50 (19.7)	63 (20.3)	
BCLC stage				0.007 *
0–A		92 (36.2)	147 (47.4))	
B, C, D		162 (63.8)	163 (52.6)	

Data expressed as mean ± standard deviation (SD) or *n* (%) * *p* < 0.05. AST: aspartate aminotransaminase, ALT: alanine aminotransaminase, ALP: alkaline phosphatase, AFP: α-fetoprotein, INR: International normalized ratio.

**Table 2:** Distribution of genotype and allele frequencies of the SNPs in healthy controls, patients with MASLD-HCC, and VIRAL-HCC.

	Healthy Controls (*n* = 180)	MASLD-HCC (*n* = 254)	VIRAL-HCC (*n* = 310)	Healthy Controls vs. MASLD-HCC		Healthy Controls vs. VIRAL-HCC		MASLD-HCC vs. VIRAL-HCC	
Polymorphisms				OR (95% CI)	*p*	OR (95% CI)	*p*	OR (95% CI)	*p*
*PNPLA3* rs738409									
Genotype frequency									
CC	83 (46.1)	86 (33.9)	126 (40.6)	1.00		1.00		1.00	
CG	76 (42.2)	107 (42.1)	136 (43.9)	0.74 (0.47–1.15)	0.153	0.85 (0.56–1.28)	0.413	1.15 (0.78–1.70)	0.455
GG	21 (11.7)	61 (24.0)	48 (15.5)	0.36 (0.19–0.66)	<0.001 *	0.66 (0.35–1.22)	0.167	1.86 (1.14–3.05)	0.009 *
CG + GG	97 (53.9)	168 (66.1)	184 (59.4)	0.59 (0.39–0.91)	0.009 *	0.80 (0.54–1.18)	0.238	1.33 (0.93–1.92)	0.098
Allele frequency									
Major (C)	0.67	0.55	0.63	1.0		1.0		1.0	
Minor (G)	0.33	0.45	0.37	0.60 (0.33–1.11)	0.08	0.84 (0.45–1.56)	0.553	1.39 (0.76–2.55)	0.250
*TM6SF2* rs58542926									
Genotype frequency									
CC	145 (80.5)	160 (63.0)	241 (77.7)	1.00		1.00		1.00	
CT	34 (18.9)	76 (29.9)	61 (19.7)	0.49 (0.30–0.80)	0.003 *	0.93 (0.56–1.51)	0.749	1.88 (1.24–2.83)	0.002 *
TT	1 (0.5)	18 (7.1)	8 (2.6)	0.06 (0.001–0.4)	<0.001 *	0.21 (0.004–1.58)	0.104	3.39 (1.36–9.21)	0.003 *
CT + TT	35 (19.4)	94 (37.0)	69 (22.2)	0.41 (0.25–0.66)	<0.001 *	0.84 (0.52–1.36)	0.463	2.05 (1.39–3.02)	<0.001 *
Allele frequency									
Major (C)	0.9	0.78	0.88	1.00		1.00		1.00	
Minor (T)	0.1	0.22	0.12	0.39 (0.16–0.94)	0.02 *	0.81 (0.29–2.18)	0.651	2.32 (1.03–5.42)	0.027 *
*HSD17B13* rs6834314									
Genotype frequency									
AA	67 (37.2)	132 (52.0)	152 (49.0)	1.00		1.00		1.00	
AG	90 (50.0)	104 (40.9)	119 (38.4)	1.70 (1.11–2.62)	0.01 *	1.71 (1.13–2.60)	0.008 *	1.01 (0.69–1.45)	0.972
GG	23 (12.8)	18 (7.1)	39 (12.6)	2.52 (1.20–5.31)	0.006 *	1.33 (0.70–2.50)	0.333	0.53 (0.27–0.97)	0.039 *
AG + GG	113 (62.8)	122 (48.0)	158 (51.0)	1.82 (1.21–2.75)	0.002 *	1.62 (1.09–2.41)	0.01 *	0.89 (0.63–1.26)	0.488
Allele frequency									
Major (A)	0.62	0.72	0.68	1.00		1.00		1.00	
Minor (G)	0.38	0.28	0.32	1.58 (0.83–2.98)	0.133	1.30 (0.69–2.43)	0.373	0.83 (0.43–1.58)	0.537

OR: odds ratio, CI: confidence interval, * *p* < 0.05.

**Table 3:** Factors associated with the overall survival of patients with HCC.

	Cases	Events	MST	Univariate	Multivariate		
Factors	564	139	51	HR (95% CI)	*p*	aHR (95%CI)	*p*
*PNPLA3* rs738409							
CC + CG	454	108	55	Ref (1.00)			
GG	110	31	45	1.14 (0.78–1.67)	0.492		
*TM6SF2* rs58542926							
CC + CT	538	129	51	Rec (1.00)			
TT	26	10	46	1.11 (0.59–2.11)	0.746		
*HSD17B13* rs6834314							
AA + AG	507	122	44	Ref (1.00)		Ref (1.00)	
GG	57	17	65	0.50 (0.29–0.85)	0.008 *	0.44 (0.26–0.75)	0.002 *
Age							
≤65	306	69	58	Ref (1.00)			
≥65	258	70	45	1.25 (0.91–1.73)	0.175		
Sex							
Female	144	35	52	Ref (1.00)			
Male	420	104	51	0.97 (0.67–1.39)	0.873		
HCC etiology							
Viral	310	58	66	Ref (1.00)		Ref (1.00)	
MASLD	254	81	42	1.58 (1.13–2.18)	0.006 *	1.48 (1.03–2.13)	0.035 *
Diabetes							
No	430	102	55	Ref (1.00)			
Yes	134	37	41	1.23 (0.85–1.77)	0.274		
Hypertension							
No	426	96	61	Ref (1.00)		Ref (1.00)	
Yes	138	43	41	1.44 (1.02–2.05)	0.039 *	1.13 (0.78–1.65)	0.517
Tumor size (cm)							
≤3.0	255	50	79	Ref (1.00)		Ref (1.00)	
≥3.0	309	89	37	2.54 (1.80–3.59)	<0.001 *	2.59 (1.81–3.73)	<0.0018 *
Extrahepatic metastasis							
No	436	92	63	Ref (1.00)		Ref (1.00)	
Yes	128	47	34	1.55 (1.09–2.19)	0.014 *	1.41 (1.01–2.02)	0.006 *
Cirrhosis							
No	122	32	44	Ref (1.00)		Ref (1.00)	
Yes	442	107	61	0.61 (0.42–0.88)	0.009 *	0.74 (0.51–1.08)	0.120
Child–Pugh score							
A	450	106	58	Ref (1.00)			
B, C	113	33	42	1.45 (1.00–2.10)	0.051		
BCLC stage							
0–A	240	41	79	Ref (1.00)		Ref (1.00)	
B C D	324	98	41	2.02 (1.42–2.87)	<0.001 *	1.37 (0.98– 2.01)	0.093

MST: Median survival time, HR: hazard ratio, aHR: adjusted hazard ratio, CI: confidence interval, * *p* < 0.05.

## Data Availability

The datasets used and analyzed during the current study are available from the corresponding author upon reasonable request.

## Supplementary Materials

The following supporting information can be downloaded at: https://www.mdpi.com/article/10.3390/genes16070808/s1, Figure S1: Effect of genetic polymorphisms on the prognosis of MASLD-HCC; Figure S2: Effect of genetic polymorphisms on the prognosis of VIRAL-HCC; Table S1: Hardy–Weinberg Equilibrium (HWE); Table S2: Factors associated with the survival of patients with MASLD-HCC; Table S3. Factors associated with the survival of patients with VIRAL-HCC.

## Abbreviations

HCC	Hepatocellular carcinoma
MASLD	Metabolic dysfunction-associated steatotic liver disease
NAFLD	Non-alcoholic fatty liver disease
BCLC	Barcelona Clinic Liver Cancer
PNPLA3	Patatin-like phospholipase domain-containing protein 3
TM6SF2	Transmembrane 6 superfamily member 2
HSD17B13	Hydroxysteroid 17-β dehydrogenase 13
HBV	Hepatitis B virus
HCV	Hepatitis C virus
GWAS	Genome-wide association study
CT	Computed tomography
MRI	Magnetic resonance imaging
OR	Odds ratio
CI	Confidence interval
OS	Overall survival
MST	Median survival time
MAF	Minor allele frequency
