# Supplementary material for: The Distribution and Survival Association of Genetic Polymorphisms in Thai Patients with Hepatocellular Carcinoma According to Underlying Liver Disease
Source: Genes (Basel). 2025 Jul 9;16(7):808. doi: 10.3390/genes16070808 (PMC12295243; doi:10.3390/genes16070808)
Supplement: Supplementary file 1 [file genes-16-00808-s001.zip › genes-3719460-supplementary.pdf]

Table S1. Hardy-Weinberg Equilibrium (HWE)

**SNPs**	**Genotype**	**Observed**	**Expected**	***p***
PNPLA3 rs738409	CC	282	271.57	0.278
	CG	335	355.85	
	GG	127	116.57	
TM6SF2 rs58542926	CC	550	547.10	0.316
	CT	176	197.23	
	TT	18	17.78	
HSD17B13 rs6834314	AA	351	346.18	0.717
	AG	313	322.64	
	GG	80	75.18	

*$p < 0.05$

Table S2. Factors associated with the survival of patients with MASLD-HCC

	Cases	Events	MST	Univariate		Multivariate	
**Factors**	**254**	**81**	**42**	**HR (95% CI)**	***p***	**aHR (95%CI)**	***p***
PNPLA3 rs738409							
CC + CG	192	64	41	Ref (1.00)			
GG	62	17	40	0.84 (0.50 – 1.39)	0.498		
TM6SF2 rs58542926							
CC + CT	236	75	61	Ref (1.00)			
TT	18	6	41	1.09 (0.47 – 2.49)	0.847		
HSD17B13 rs6834314							
AA + AG	236	72	37	Ref (1.00)		Ref (1.00)	
GG	18	9	64	0.39 (0.19 – 0.84)	0.016*	**0.38 (0.18 – 0.81)**	**0.011***
Age							
≤ 65	91	28	44	Ref (1.00)			
≥ 65	163	53	41	1.11 (0.71 -1.73)	0.654		
Sex							
Female	75	20	52	Ref (1.00)			
Male	179	61	41	1.34 (0.81 – 2.19)	0.251		
Diabetes							
No	156	47	45	Ref (1.00)			
Yes	98	34	37	1.20 (0.78 – 1.85)	0.407		
Hypertension							
No	156	43	44	Ref (1.00)			
Yes	98	38	38	1.26 (0.82 -1.93)	0.287		
Tumor size (cm)							
≤ 3.0	128	32	63	Ref (1.00)		Ref (1.00)	
≥ 3.0	126	49	27	2.63 (1.68 – 4.09)	<0.001*	**2.68 (1.72 – 4.19)**	**<0.001***
Extrahepatic metastasis							
No	167	50	45	Ref (1.00)			
Yes	87	31	32	1.19 (0.77 – 1.87)	0.431		
Cirrhosis							
No	72	22	44	Ref (1.00)			
Yes	182	57	41	0.71 (0.45 – 1.13)	0.152		
Child-pugh score							
A	204	66	41	Ref (1.00)			
B, C	50	15	45	0.93 (0.53 – 1.63)	0.811		
BCLC stage							
0 - A	92	20	62	Ref (1.00)			
B C D	162	61	41	1.57 (0.96 – 2.57)	0.074		

**MST*: Median Survival Time, *HR*: hazard ratio, *aHR*: adjusted hazard ratio, *CI*: confidence interval, $p < 0.05$

Table S3. Factors associated with the survival of patients with VIRAL-HCC

	Cases	Events	MST	Univariate		Multivariate	
**Factors**	**310**	**58**	**66**	**HR (95% CI)**	***p***	**aHR (95%CI)**	***p***
PNPLA3 rs738409							
CC + CG	262	44	66	Ref (1.00)			
GG	48	14	41	1.61 (0.91 – 2.84)	0.101		
TM6SF2 rs58542926							
CC + CT	302	54	66	Ref (1.00)			
TT	8	8	51	1.07 (0.39– 2.97)	0.893		
HSD17B13 rs6834314							
AA + AG	271	50	66	Ref (1.00)			
GG	39	8	65	0.56 (0.26 – 1.24)	0.152		
Age							
≤ 65	215	41	65	Ref (1.00)			
≥ 65	95	17	58	1.00 (0.58 – 1.72)	0.998		
Sex							
Female	69	15	51	Ref (1.00)			
Male	241	43	66	0.72 (0.42 – 1.24)	0.241		
Diabetes							
No	274	55	65	Ref (1.00)			
Yes	36	3	86	0.59 (0.21 – 1.59)	0.292		
Hypertension							
No	270	53	66	Ref (1.00)			
Yes	40	5	41	1.14 (0.52 -2.52)	0.741		
Tumor size (cm)							
≤ 3.0	113	18	86	Ref (1.00)		Ref (1.00)	
≥ 3.0	197	40	50	2.68 (1.54 – 4.68)	0.001*	**2.10 (1.10 – 4.04)**	**0.025***
Extrahepatic metastasis							
No	269	42	79	Ref (1.00)		Ref (1.00)	
Yes	41	16	36	1.88 (1.05 – 3.35)	0.033*	1.59 (0.89 – 2.87)	0.116
Cirrhosis							
No	50	8	40	Ref (1.00)			
Yes	260	50	66	0.59 (0.32 – 1.13)	0.111		
Child-pugh score							
A	246	40	79	Ref (1.00)		Ref (1.00)	
B, C	63	18	38	2.32 (1.38 – 3.88)	0.001*	**2.18 (1.29 – 3.66)**	**0.003***
BCLC stage							
0 - A	148	21	86	Ref (1.00)		Ref (1.00)	
B C D	162	37	50	2.22 (1.33 – 3.72)	0.002*	1.39 (0.76 – 2.53)	0.287

**MST*: Median Survival Time, *HR*: hazard ratio, *aHR*: adjusted hazard ratio, *CI*: confidence interval, $p < 0.05$

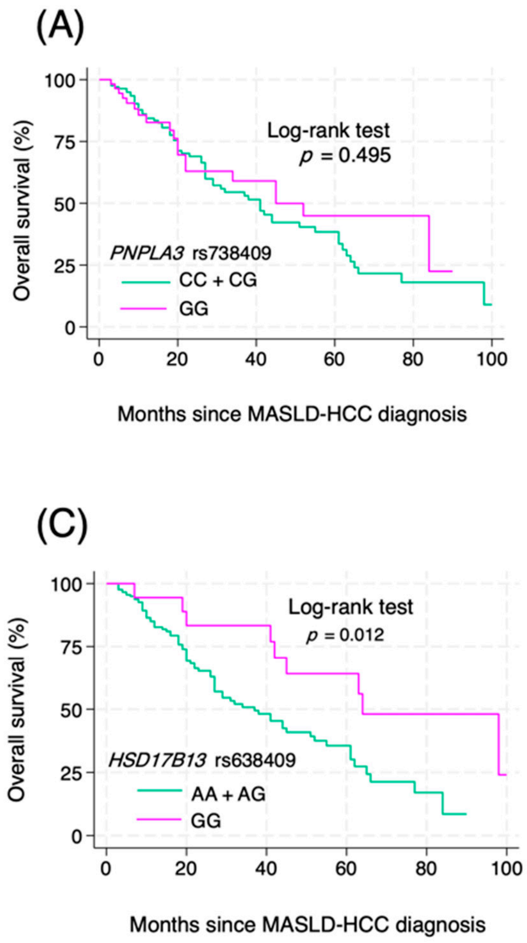

Figure S1. Effect of genetic polymorphisms on the prognosis of MASLD-HCC*:*
(A) *PNPLA3* rs738409 CC + CG vs GG; (B) *TM6SF2* rs58542926 CC+CT vs TT;(C) *HSD17B13* rs6834314 AA+AG vs GG

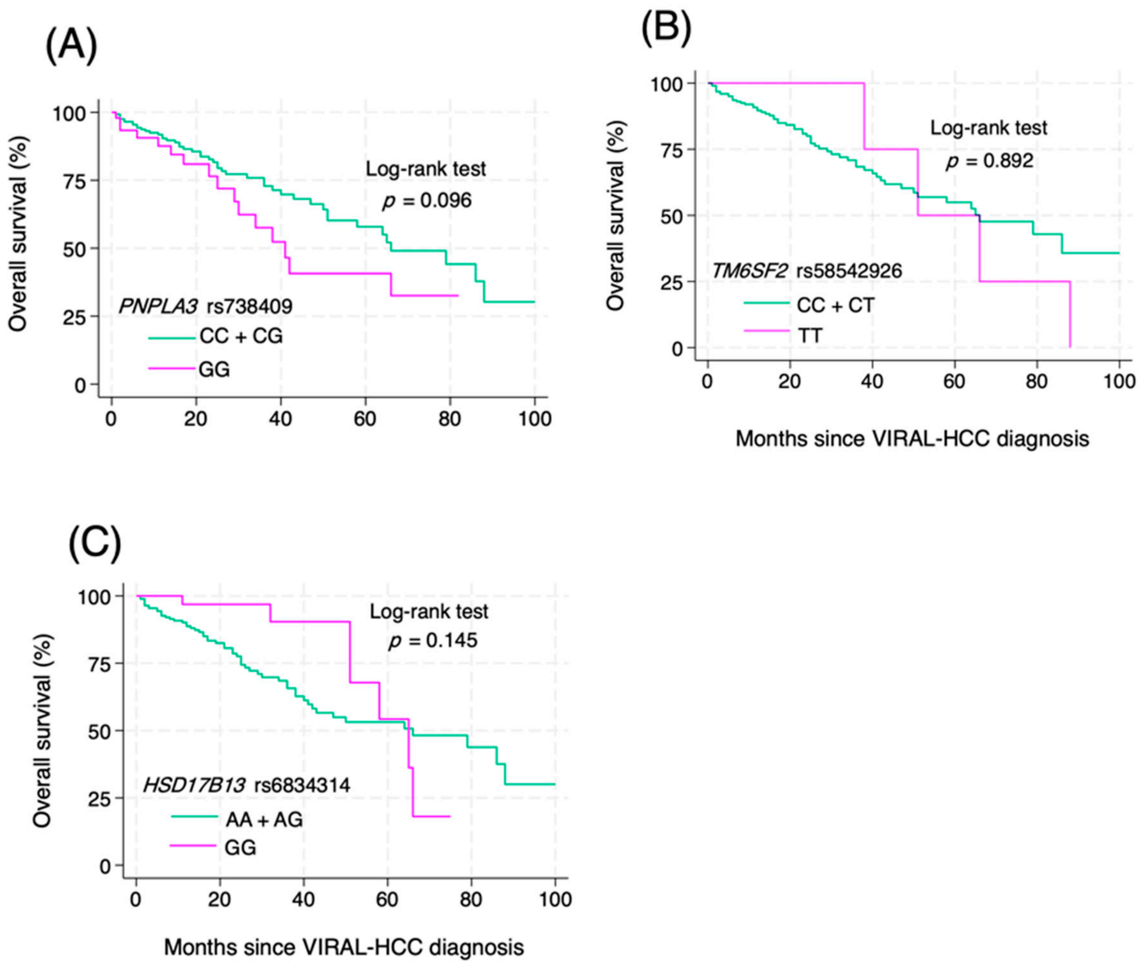

Figure S2. Effect of genetic polymorphisms on the prognosis of VIRAL-HCC*:*
(A) *PNPLA3* rs738409 CC + CG vs GG; (B) *TM6SF2* rs58542926 CC+CT vs TT;(C) *HSD17B13* rs6834314 AA+AG vs GG
